# Cytological Diagnosis of Hepatic Metastasis from Rectal Gastrointestinal Stromal Tumor: A Case Report

**DOI:** 10.15190/d.2025.1

**Published:** 2025-03-31

**Authors:** Gauri Niranjan, Pallavi Prasad, Archana Verma, Ashok Kumar

**Affiliations:** ^1^Department of Pathology, Sanjay Gandhi Postgraduate Institute of Medical Sciences, Lucknow, India; ^2^Department of Surgical Gastroenterology, Sanjay Gandhi Postgraduate Institute of Medical Sciences, Lucknow, India

**Keywords:** Cytology, gastrointestinal stromal tumour (GIST), liver, mesenchymal, metastasis, rectal.

## Abstract

Gastrointestinal stromal tumours (GIST) are rare mesenchymal tumours which represent 1% to 3% of all gastrointestinal neoplasms. Rectal location of GIST is extremely rare accounting for 5% of GIST and only 0.1% of rectal tumours. They usually metastasise to the liver (65%). We hereby report a case of rectal stromal tumour with hepatic metastasis. A 55-year-old female presented with pelvic pain, associated with rectal bleeding. A thoracoabdominal computed tomography showed a large heterogeneous enhancing mass, arising from the rectum, anal canal and distal sigmoid colon measuring 12.3x8.7x7.6cm. Based on histopathological examination followed by immunohistochemistry, she was diagnosed with locally advanced rectal GIST. The tumour reduced in size after neoadjuvant-targeted treatment with imatinib. A local resection of the rectal GIST was successfully performed, and a diversion colostomy was done, later colostomy bag was attached. Following the operation, oral imatinib treatment was continued. On subsequent follow-up, her triple phase CECT whole abdomen showed multiple small well-defined peripherally enhancing hypodense liver lesions, the largest measuring 29x18mm suggestive of metastases. Ultrasound-guided fine needle aspiration from a liver lesion was reported as metastatic GIST. The patient underwent surgery, sunitinib was started and was discharged in stable condition. Thus, cytologic examination provides rapid interpretation, is a less invasive technique than open biopsy, and provides a cost-effective modality for diagnosing and managing inaccessible lesions.

## INTRODUCTION

Gastrointestinal stromal tumour (GIST) is the most frequent mesenchymal tumour that occurs in the gastrointestinal tract accounting for 1% to 3% of all the gastrointestinal tract neoplasm. GIST is a rare tumour and is a specific mesenchymal neoplasm of the gastrointestinal tract which expresses CD117, a c-kit proto-oncogene protein, and shows a gain of function mutation of c-kit gene that encodes a growth factor receptor with tyrosine kinase activity ^1^. It accounts for 1% to 3 % of gastric cancer ^2^. GIST is particularly common in the stomach (55%) followed by the colon ^3^. Rectum is an uncommon site for GIST. It accounts for 5% of the GIST. It accounts for 5% of GISTs and only 0.1% of the rectal cancers ^4^. About 30% of the GISTs are malignant and the liver is the most common site for metastasis ^5,6,7,8^. The criteria for differentiation of benign from malignant GIST remain controversial. Many parameters have been proposed, and tumour size and proliferative activity are the most important prognostic indicators ^9,10^. It is reported that approximately two-thirds of the patients with recurrence have liver metastasis (65%). We studied the cytomorphological features in a case of rectal GIST presenting with liver metastasis.

## CASE REPORT

**Figure 1:**
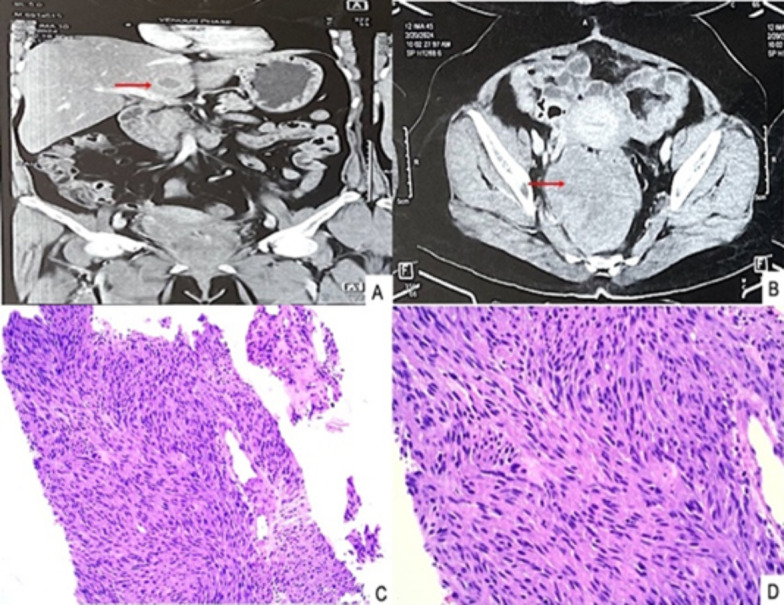
Figure 1. Large heterogenous enhancing mass, arising from the rectum, anal canal and distal sigmoid colon. (A): CT whole abdomen showing the largest liver lesion measuring 29x18mm. (B): CT abdomen and pelvis showing a heterogeneous enhancing mass, arising from the rectum, anal canal and distal sigmoid colon. (C, D): Section from rectal biopsy showing a mesenchymal tumour composed of long and short interlacing fascicles and a storiform pattern of arrangement of spindle tumour cells. These tumour cells display mildly pleomorphic round to elongated nuclei, granular chromatin, inconspicuous nucleoli and a moderate amount of eosinophilic cytoplasm (H&E stain).

A 55-year-old female was admitted to the surgical gastroenterology department of our institute with complaints of pelvic pain, associated with rectal bleeding for the last two and a half months. She also gave a history of constipation and weight loss for the last six months. There was no history of nausea, vomiting or hesitancy in voiding urine. Physical examination revealed mild pallor and a rectal mass palpable during digital rectal examination. Colonoscopy revealed a mass in the mid-distal rectum, firm, exophytic and fixed in nature, located at 4 cm from the anal verge, with mild circumferential narrowing. Overlying mucosa was intact and appeared slightly erythematous with no ulceration. A thoracoabdominal computed tomography showed a large heterogenous enhancing mass, arising from the rectum, anal canal and distal sigmoid colon measuring 12.3 x8.7 x7.6cm (Figure 1). At her first visit, she was diagnosed with locally advanced rectal GIST. Histopathological examination and subsequent immunohistochemistry confirmed the diagnosis. On histology, the rectal biopsy showed a mesenchymal tumour composed of long and short interlacing fascicles and a storiform pattern of arrangement of spindle-shaped tumour cells. These tumour cells display mild pleomorphic round to elongated nuclei, granular chromatin, inconspicuous nucleoli and a moderate amount of eosinophilic cytoplasm. Frequent mitotic activity was present (>5/50hpf). No necrosis was identified.

**Figure 2:**
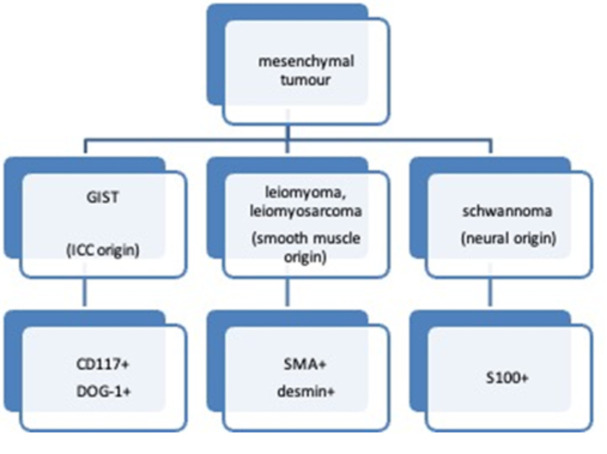
FIgure 2. Algorithm for immunohistochemical panel applied in the present case.

Based on these findings, differential diagnoses were smooth muscle tumours and neurogenic tumours. For further confirmation and typing, immunohistochemistry was applied, on which the tumour cells were diffusely positive for CD117, DOG1 (markers for GIST) and negative for smooth muscle actin (SMA), desmin (markers for smooth muscle origin) and S100 (marker of neural origin) (Figure 2).

**Figure 3:**
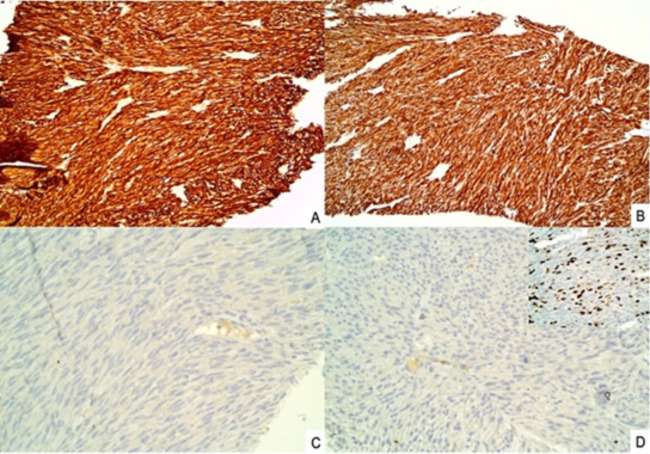
Figure 3. Tumour cells are diffusely positive for (A) DOG-1, (B) CD117 and negative for (C) S-100 and (D) SMA. ki67 index (inset) is 10%.

Ki67 proliferation index was ~ 10% (Figure 3). The patient underwent neoadjuvant targeted chemotherapy with imatinib, following which local resection of the rectal GIST was successfully performed and diversion colostomy was done. Following the operation, oral imatinib treatment was continued. After 1 year patient again presented to our institute with complaints of anorexia and weight loss.

**Figure 4:**
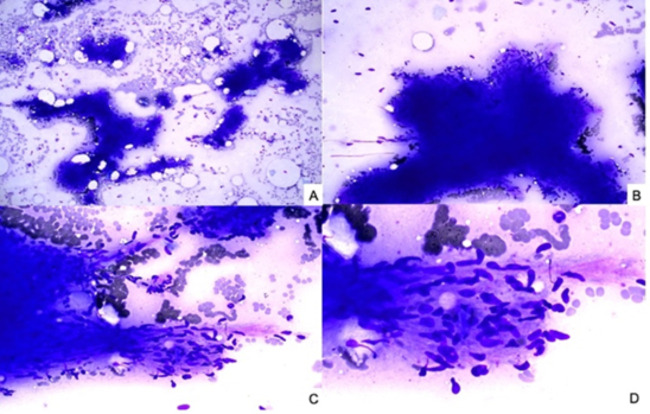
Figure 4. Nuclei morphology (A-D) Cytology smears from liver lesion showing fragments, clusters as well as singly scattered spindle-shaped cells disposed on a blood-mixed background. These tumour cells are spindle-shaped with tapering nuclei, fine chromatin, inconspicuous nucleoli and poorly defined cytoplasm (MGG stain).

A triple-phase CECT whole abdomen showed multiple small well-defined peripherally enhancing hypodense lesions in the liver, the largest measuring 29x18mm, suggestive of metastases. It also showed post-operative changes with large well-defined heterogeneously enhancing mass-lesion in the presacral space likely arising from the posterior wall of the rectum and anorectal junction with mass effect and extension, suggestive of residual lesion. Ultrasound-guided fine-needle aspiration (FNA) smears from liver SOL were cellular and showed fragments, clusters as well as singly scattered spindle-shaped cells disposed on a hemorrhagic background admixed with benign hepatocytes. Myxoid stroma was noted in the background of these cell clusters and fragments. These cells were spindle-shaped with tapering nuclei, fine chromatin, inconspicuous nucleoli and poorly defined cytoplasm (Figure 4). Necrosis or mitosis was not seen. The case was consequently reported as metastatic GIST. R0 resection of the liver metastasis was achieved. After the operation, sunitinib was started. The patient was thereafter discharged and is presently doing well on regular follow-up.

## DISCUSSIONS

GISTs which arise from the intestinal cell of Cajal or its precursor, the intestinal mesenchymal precursor cell, are the most common mesenchymal neoplasm of the gastrointestinal tract ^11^. GISTs exhibit biological characteristics ranging from benign to clearly malignant. The incidence of rectal GISTs (accounting for 4% to 6% of all GISTs) is lower than that of the colonic GIST. Most rectal GISTs are located in the middle and lower rectum ^12^. The onset of the rectal GIST is vague and uncertain and is related to the size, location, and extent of the tumour. The main symptom is a change in bowel habits and some patients may have bloody stools. The lesions are often found incidentally during health check-ups. Since the introduction of EUS-guided FNA, the primary modality for the diagnosis of GIST and metastatic GIST is mainly FNA^13,14^. The cytology of GIST can often overlap with other spindle cell neoplasms including schwannoma, leiomyoma, leiomyosarcoma and epithelioid leiomyoblastoma ^15^. Differential diagnosis between GISTs and gastrointestinal leiomyomas is difficult due to overlapping clinical andcytology. Leiomyomas exhibit variable cellularity, consisting of soft spindle-shaped cells with abundant cytoplasm, often with a fibrillary appearance. No atypia, mitosis, or epithelioid cells are seen. Gastrointestinal leiomyosarcoma is less common than leiomyoma. GIST and leiomyosarcoma may have a spindle or epithelioid cell morphology. Leiomyosarcomas show three-dimensional, tightly adherent, sharp-edged spindle cell syncytia, often with nuclear extrusion artefact. Leiomyosarcoma often shows pleomorphism. Epithelioid cell morphology, mitosis and necrosis are occasionally observed in both tumour types. Benign and malignant nerve sheath tumours show fibrillary cytoplasm and wavy nuclei resembling a distinct nerve cell and features such as nuclear palisading may be focal ^15^. Cytology is very useful for preoperative diagnosis of hepatic lesions that may otherwise be inaccessible or carry significant risks for biopsy-related complications. In addition, cytology results have a shorter turnaround time and are inexpensive as compared to histopathology and imaging techniques ^16^. Histopathological examination has a higher turnaround time poses procedure-related complications and is comparatively expensive. Computed tomography (CT) is the primary modality used for the initial diagnosis of GISTs, surgical planning, postsurgical surveillance, monitoring therapy response and detecting metastases. MRI provides findings similar to CT and additional parameters like apparent diffusion coefficient, degree of enhancement, and perfusion parameters. It can also help diagnose hepatic metastasis, which may be missed on CT ^17.^Definitive diagnosis of GIST is based on a combination of histology and immunohistochemical staining criteria. GIST are typically immunoreactive for c-KIT. c-KIT (CD117) is a transmembrane receptor that is part of the tyrosine kinase receptor complex. CD117 positivity is seen in about 90-100% of GIST while positivity for CD34, the hematopoietic progenitor cell antigen, is reported in 70-80% of GIST. On histology, tumour cells are disposed in interlacing fascicles and storiform pattern of spindle cells. These tumour cells display moderately pleomorphic round to elongated nuclei, granular chromatin, conspicuous nucleoli and a moderate amount of cytoplasm. The c-kit(CD117) and/or DOG1 expression is essential to diagnose GIST.The only treatment for intestinal stromal tumours is surgery. The most common site of metastasis is liver. There are no specific findings for GIST on CT ^18^. Imatinib is used as part of adjuvant therapy or in replacement form. The prognosis has improved, especially since the introduction of imatinib as adjuvant therapy, but the metastatic prognosis is poor ^19^. The 5-year disease-free survival rate after metastatic GIST resection is approximately 43.9%^20^.

### Conclusion

The incidence of rectal GIST is low and the clinical site is nonspecific. They usually metastases to the liver (65%) followed by the peritoneal surface (50%). Cytology is a rapid and cost-effective technique for diagnosing and monitoring GISTs. This case report highlights the utility of fine needle aspiration cytology as a useful tool in rendering an accurate and timely diagnosis; especially in this scenario where biopsy was inaccessible, thus guiding prompt patient therapy. Patients' survival and quality of life can be improved through comprehensive management and periodic monitoring.
